# Independent Regulation of Reovirus Membrane Penetration and Apoptosis by the μ1 ϕ Domain

**DOI:** 10.1371/journal.ppat.1000248

**Published:** 2008-12-26

**Authors:** Pranav Danthi, Caroline M. Coffey, John S. L. Parker, Ty W. Abel, Terence S. Dermody

**Affiliations:** 1 Department of Pediatrics, Vanderbilt University School of Medicine, Nashville, Tennessee, United States of America; 2 Elizabeth B. Lamb Center for Pediatric Research, Vanderbilt University School of Medicine, Nashville, Tennessee, United States of America; 3 Baker Institute for Animal Health, College of Veterinary Medicine, Cornell University, Ithaca, New York, United States of America; 4 Department of Pathology, Vanderbilt University School of Medicine, Nashville, Tennessee, United States of America; 5 Department of Microbiology and Immunology, Vanderbilt University School of Medicine, Nashville, Tennessee, United States of America; University of North Carolina, United States of America

## Abstract

Apoptosis plays an important role in the pathogenesis of reovirus encephalitis. Reovirus outer-capsid protein μ1, which functions to penetrate host cell membranes during viral entry, is the primary regulator of apoptosis following reovirus infection. Ectopic expression of full-length and truncated forms of μ1 indicates that the μ1 ϕ domain is sufficient to elicit a cell death response. To evaluate the contribution of the μ1 ϕ domain to the induction of apoptosis following reovirus infection, ϕ mutant viruses were generated by reverse genetics and analyzed for the capacity to penetrate cell membranes and elicit apoptosis. We found that mutations in ϕ diminish reovirus membrane penetration efficiency by preventing conformational changes that lead to generation of key reovirus entry intermediates. Independent of effects on membrane penetration, amino acid substitutions in ϕ affect the apoptotic potential of reovirus, suggesting that ϕ initiates apoptosis subsequent to cytosolic delivery. In comparison to wild-type virus, apoptosis-defective ϕ mutant viruses display diminished neurovirulence following intracranial inoculation of newborn mice. These results indicate that the ϕ domain of μ1 plays an important regulatory role in reovirus-induced apoptosis and disease.

## Author Summary

Many viruses produce central nervous system disease by infecting and systematically killing host neurons via a process known as programmed cell death, or apoptosis. However, for most viruses, it is not understood how viral invasion is detected by host cells nor how this recognition triggers a signaling cascade that leads to apoptotic cell death. We used mammalian reovirus to understand the intricacies of this virus–host interaction. We found that the ϕ domain of reovirus membrane-penetration protein μ1 mediates two functions during the reovirus replication cycle. First, ϕ affects the efficiency with which reovirus breaches the host cell membrane barrier. Second, ϕ controls the strength of the prodeath signal via its subcellular localization or its interaction with a cellular sensor that connects reovirus to the cell death machinery. Importantly, these effects are independent, as judged by analysis of mutants displaying discordant alterations in membrane penetration and apoptosis. Remarkably, we found that the capacity of reovirus to induce apoptosis affects its capacity to cause disease. These findings provide insight into mechanisms by which viruses activate signaling networks that contribute to cell death and suggest that the host–pathogen death-signaling interface is a viable target for therapy against viruses that afflict the central nervous system.

## Introduction

Neurological disease is one of the most serious manifestations of viral infection. A diverse group of neurotropic viruses including alphaviruses, bunyaviruses, flaviviruses, herpesviruses, and rhabdoviruses are capable of causing encephalitis. Central nervous system (CNS) disease following infection by many of these viruses is associated with neuronal apoptosis [1]–[5]. Identification of viral components that engage the cellular machinery to evoke apoptosis is prerequisite to the development of novel therapeutic targets against encephalitic viruses. However, limited information exists about this critical pathogen-host interface.

Mammalian reoviruses are highly tractable models for analysis of virus-host interactions. Studies using these viruses have provided significant insights into mechanisms by which viruses initiate proapoptotic signaling responses that contribute to the pathogenesis of viral encephalitis. Following infection of newborn mice, reoviruses disseminate systemically, producing injury to a variety of organs, including the CNS, heart, and liver [6]. Infection of mice with type 3 reovirus results in fatal encephalitis [7]–[9], which is associated with extensive apoptosis at sites of viral replication [10]–[12]. Modulation of apoptosis in infected animals using pharmacological inhibitors or through genetic means attenuates CNS disease, highlighting an important contributory role of apoptosis to the pathogenesis of encephalitis [11]–[14].

In cultured cells, reovirus-induced apoptosis does not require de novo synthesis of viral RNA and protein [15],[16], indicating that the proapoptotic stimulus is contained within infecting viral capsids. Consistent with these findings, strain-specific differences in the capacity of reovirus to induce apoptosis segregate genetically with the viral S1 and M2 gene segments [17]–[19], which encode attachment protein σ1 and outer-capsid protein μ1, respectively [20],[21]. Studies in which σ1 attachment to its cognate receptors, junctional adhesion molecule-A (JAM-A) and sialic acid, was uncoupled from viral disassembly by providing an alternative means of cell entry indicate that signaling pathways triggered by σ1-receptor interactions are dispensable for reovirus-induced apoptosis [16]. Regardless of the receptors used to mediate attachment, initiation of prodeath signaling following reovirus infection requires viral disassembly in cellular endosomes [16]. Concordantly, these studies identified the viral M2 gene segment as the primary determinant of strain-specific differences in reovirus apoptosis [16], suggesting an essential function for the μ1 protein in apoptosis induction.

**Figure 1:**
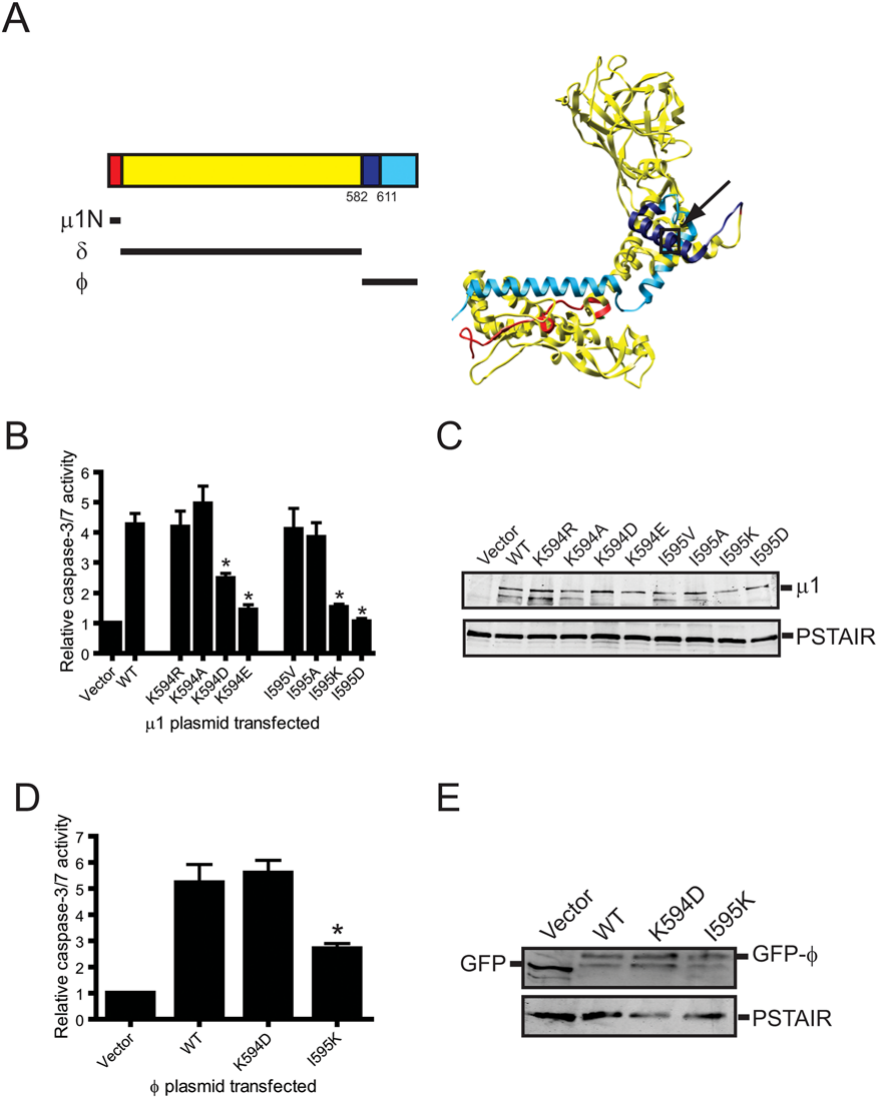
Charge substitutions of μ1 ϕ residues Lys594 and Ile595 diminish apoptosis-inducing capacity of ectopically expressed μ1 and ϕ. (A) Crystal structure of a μ1 monomer (PBD∶1JMU) [36]. The μ1N, δ, and ϕ domains are indicated in red, yellow, and blue, respectively. The location of a minimal functional region of ϕ (spanning amino acids 582–611) sufficient for apoptosis induction following expression in cells [33] is indicated in dark blue. A black box indicates the position of Lys594 and Ile595. (B) 293T cells were transfected with a plasmid expressing either wild-type or the indicated point-mutant μ1 protein. Caspase-3/7 activity in cell lysates was quantified 24 h post-transfection. Results are expressed as the mean ratio of caspase-3/7 activity from transfected cell lysates to that from empty vector-transfected cells for triplicate samples. Error bars indicate SD. *, *P*<0.05 as determined by Student's *t*-test in comparison to cells infected with wild-type μ1. (C) 293T cells treated with Z-VAD-FMK were transfected with a plasmid expressing either wild-type or the indicated point-mutant μ1 protein. Cell lysates were prepared at 24 h post-transfection, resolved by SDS-PAGE, and immunoblotted using a MAb specific for μ1 (upper panel). As a control for protein concentration, the blots also were probed with an antibody specific for the PSTAIR peptide of Cdk1 (lower panel). (D) 293T cells were transfected with a plasmid expressing either wild-type, K594D, or I595K GFP-tagged ϕ protein. Caspase-3/7 activity in cell lysates was quantified 24 h post-transfection. Results are expressed as the mean ratio of caspase-3/7 activity from transfected cell lysates to that from empty vector-transfected cells for triplicate samples. Error bars indicate SD. *, *P*<0.05 as determined by Student's *t*-test in comparison to cells infected with wild-type μ1. (E) 293T cells treated with Z-VAD-FMK were transfected with a plasmid expressing either wild-type, K594D, or I595K GFP-tagged ϕ protein. Cell lysates were prepared at 24 h post-transfection, resolved by SDS-PAGE, and immunoblotted using a MAb specific for GFP (upper panel). As a control for protein concentration, the blots also were probed with an antibody specific for the Cdk1 PSTAIR peptide (lower panel).

In addition to its structural and proapoptotic functions, the μ1 protein plays an essential role in penetration of host cell membranes during reovirus cell entry. Following uptake into cellular endosomes, reovirus undergoes proteolytic disassembly resulting in removal of outer-capsid protein σ3 and cleavage of the μ1 protein to form the δ and ϕ fragments (Figure 1A), which remain associated with the newly generated infectious subvirion particles (ISVPs) [22]–[25]. Conformational changes within the ISVP-associated δ fragment lead to formation of ISVP*s and allow the autocatalytic cleavage and release of a small myristoylated N-terminal fragment of μ1, μ1N (Figure 1A) [26]–[30]. The released μ1N fragment is thought to function alone or act in concert with the lipophilic ISVP*s to form pores in host cell membranes that release the viral core into the cytoplasm to initiate infection [31],[32].

Introduction of single amino acid substitutions into the δ region of μ1 decreases the capacity of the resultant mutant viruses to effect membrane penetration, mobilize proapoptotic transcription factors, NF-κB and IRF-3, and evoke apoptosis [12]. These findings suggest that the membrane-penetration and apoptosis-induction functions of μ1 are linked and that the δ region of μ1 is an essential modulator of both processes. However, the precise mechanism by which μ1-mediated membrane penetration activates prodeath signaling is not clear. It is possible that membrane penetration regulated by δ or the physiologic consequences of membrane rupture initiate proapoptotic signals. Alternatively, membrane penetration might allow delivery of the μ1 cleavage fragments into the cytoplasm where prodeath signaling is elicited. In support of the latter possibility, plasmid-driven expression of the μ1 C-terminal cleavage fragment, ϕ, in the cytoplasm is sufficient to induce apoptosis [33]. Therefore, we hypothesize that membrane penetration by reovirus results in the delivery of ϕ into the cytosol where it activates a cellular trigger to induce apoptosis.

In this study, we evaluated the role of the μ1 ϕ domain in the reovirus replication cycle by characterizing viruses with amino acid substitutions within ϕ. We found that the μ1 ϕ domain regulates both membrane penetration and apoptosis induction following reovirus infection. Analogous to mutations in δ, which impair membrane penetration by diminishing the efficiency of conformational changes in μ1 that accompany the ISVP-to-ISVP* transition [12],[28],[34], mutations in ϕ also affect membrane penetration by affecting ISVP* formation. Additionally, our studies indicate that while mutations in ϕ modulate prodeath signaling following reovirus infection, the membrane-penetration and apoptosis-inducing functions of ϕ can be genetically uncoupled, indicating that initiation of apoptosis occurs subsequent to membrane penetration. Collectively, these findings highlight an important role for the μ1 ϕ domain in both membrane penetration and apoptosis induction following reovirus infection and enhance a mechanistic understanding of how these two processes are regulated.

## Results

### Apoptosis-inducing capacity of ectopically expressed μ1 is regulated by amino acid residues 594 and 595

Studies using ectopically expressed μ1 indicate that residues 582–611 within the μ1 C-terminal cleavage fragment, ϕ, are sufficient to induce apoptosis [33]. To better inform selection of functionally important residues for the generation of mutant viruses, we constructed plasmids that express mutant μ1 proteins with amino acid substitutions within the minimal functional domain of ϕ (residues 582–611) and assessed the capacity of these constructs to stimulate the activity of effector caspases following transfection of host cells. For these initial studies, we mutagenized residues within this region predicted to either influence its helical nature or modulate its interaction with membranes. This genetic screen revealed that mutations of ϕ residues Lys594 and Ile595 substantially diminished apoptosis induction (Figure 1A and data not shown). To understand the role of these residues in evoking apoptosis, we assessed the apoptosis-inducing capacity of plasmids harboring a variety of substitutions at Lys594 and Ile595 (Figure 1B). In comparison to an empty vector control, transfection of 293T cells with a plasmid expressing wild-type μ1 resulted in ∼4-fold increase in caspase-3/7 activity. While substitution of the basic amino acid, lysine at position 594 in μ1 with an alanine or arginine did not affect apoptosis efficiency of the mutant μ1 protein, introduction of either aspartic acid or glutamic acid at that position diminished apoptotic potential. Similarly, while substitution of the hydrophobic amino acid isoleucine at position 595 with either alanine or valine did not affect its capacity to stimulate effector caspase activity, substitution with either lysine or aspartic acid significantly decreased the capacity of mutant μ1 to stimulate a cell-death response. Steady-state levels of wild-type and mutant μ1 proteins were equivalent (Figure 1C), suggesting that differences in the capacity of μ1 mutants to mediate activation of effector caspases are not related to differences in the expression or stability of the μ1 proteins. Thus, Lys594 and Ile595 influence the induction of apoptosis following μ1 expression in cells.

Since ectopically expressed μ1 is not cleaved to generate ϕ (data not shown), it is possible that substitutions at Lys594 and Ile595 affect the capacity of μ1 to induce apoptosis through indirect effects on protein conformation. To evaluate this possibility, we assessed the apoptotic potential of plasmids expressing wild-type, K594D, or I595K GFP-tagged ϕ (Figure 1D). Consistent with our previous findings [33], expression of wild-type GFP-ϕ resulted in ∼5-fold increase in effector caspase activity. We found that while a K594D substitution in GFP-ϕ did not affect its capacity to stimulate effector caspase activity, an I595K substitution significantly diminished the capacity of GFP-ϕ to induce apoptosis (Figure 1D). Since these effects do not parallel differences in the steady-state levels of GFP-tagged ϕ (Figure 1E), these results, along with findings made using full-length μ1 constructs, indicate that substitutions at Lys594 and Ile595 regulate μ1-mediated apoptosis induction via distinct mechanisms. Whereas K594D likely regulates the apoptosis induction function of μ1 by affecting μ1 conformation, I595K appears to influence the apoptotic potential of mutant μ1 proteins as a direct consequence of alterations in physicochemical properties of ϕ.

### Reovirus ϕ mutants are viable and display no growth defects

**Figure 2:**
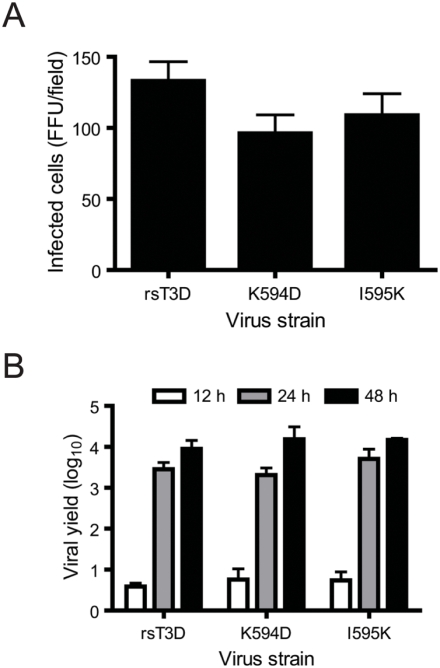
Infectivity and growth of μ1 ϕ mutants. (A) L929 cells were adsorbed with rsT3D or the indicated ϕ mutant at an MOI of 10^4^ particles/cell. After incubation at 37°C for 18 h, cells were fixed using methanol and visualized by immunostaining. Reovirus-infected cells were quantified by counting fluorescent cells. Results are expressed as mean fluorescent focus units (FFU) per field for triplicate samples. Error bars indicate SD. (B) L929 cells were adsorbed with rsT3D or the indicated ϕ mutant at an MOI of 2 PFU/cell. Titers of virus in cell lysates at the indicated intervals post-infection were determined by plaque assay. Results are expressed as viral yield for triplicate samples. Error bars indicate SD.

To evaluate the function of residues 594 and 595 in apoptosis induction in the context of virus infection, we used reverse genetics [35] to rescue wild-type recombinant strain type 3 Dearing (rsT3D) and ϕ mutant viruses, K594D or I595K, which we hypothesized to be altered in prodeath signaling (Figure 1). Viruses with these substitutions in ϕ were rescued with equivalent efficiency (data not shown). To determine whether substitutions in ϕ affect reovirus infectivity, we quantified the number of L929 cells expressing nascent viral protein following infection with rsT3D and the mutant viruses (Figure 2A). We found that infection with either rsT3D or the ϕ mutants resulted in an approximately equal number of infected cells. These data suggest that mutations in ϕ do not affect the efficiency with which reovirus establishes infection of host cells. To evaluate whether these mutations affect the capacity of reovirus to efficiently replicate in host cells, we infected L929 cells with either rsT3D or the ϕ mutant viruses at a multiplicity of infection (MOI) of 2 plaque forming units (PFU)/cell and quantified viral titer at 0, 12, 24, and 48 h following infection (Figure 2B). At 24–48 h post-infection, each of the viruses produced ∼1000- to 10 000-fold increases in infectious progeny. In concordance with these findings, no differences were detected in the replication kinetics of rsT3D, K594D, and I595K when infection was initiated at an MOI of 0.01 PFU/cell (data not shown). These data indicate that mutations at ϕ residues 594 or 595 do not compromise viral replication kinetics or yield in cell culture.

### Mutations in ϕ affect reovirus membrane-penetration efficiency

**Figure 3:**
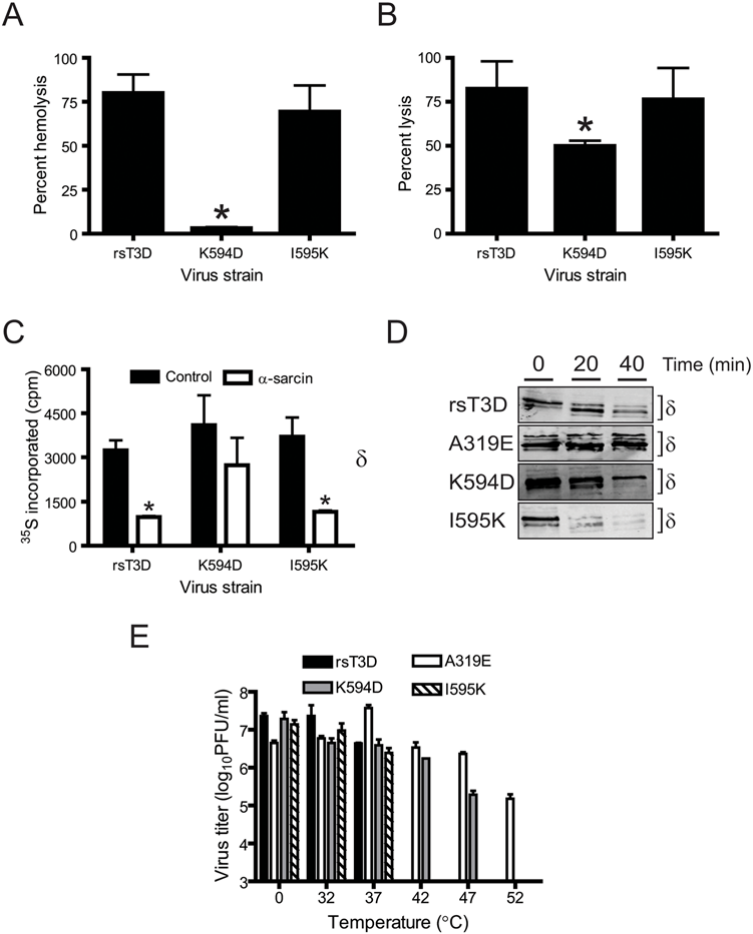
K594D exhibits altered capacity for membrane penetration. (A) A 3% v/v solution of bovine erythrocytes was incubated with 5.4×10^10^ ISVPs of rsT3D or the indicated ϕ mutant at 37°C for 1 h. Hemolysis was quantified by determining absorbance of the supernatant at 415 nm. Hemolysis following treatment of an equal number of cells with virion-storage buffer or virion-storage buffer containing 1% TX-100 was considered to be 0 or 100%, respectively. Results are expressed as mean percent hemolysis for triplicate samples. Error bars indicate SD. *, *P*<0.05 as determined by Student's *t*-test in comparison to rsT3D. (B) L929 cells preincubated with ^51^Cr-labeled sodium chromate were adsorbed with 10^5^ ISVPs/cell of rsT3D or the indicated ϕ mutant at 4°C for 1 h and incubated at 37°C for 4 h following addition of complete medium. The amount of ^51^Cr released into the medium was determined by liquid scintillation. ^51^Cr release following treatment of an equal number of cells with virion-storage buffer or by addition of 4% TX-100 to the medium was considered to be 0 or 100%, respectively. Results are expressed as mean percent lysis for triplicate samples. Error bars indicate SD. *, *P*<0.05 as determined by Student's *t*-test in comparison to rsT3D. (C) HeLa cells starved of cysteine and methionine were adsorbed with 10^6^ ISVPs/cell of rsT3D or the indicated ϕ mutant at 4°C for 1 h. Infection was initiated in medium containing ^35^S-labeled cysteine and methionine in the presence or absence of α-sarcin. Cells were lysed following incubation at 37°C for 1 h. Proteins were precipitated with TCA, and acid-precipitable radioactivity was quantified by scintillation counting. Results are expressed as mean ^35^S incorporated for triplicate samples. Error bars indicate SD. *, *P*<0.05 as determined by Student's *t*-test in comparison to ^35^S incorporated in the absence of α-sarcin. (D) CsCl-treated ISVPs of rsT3D or the indicated μ1 mutant were incubated with trypsin at 4°C for the intervals shown. Samples were resolved by SDS-PAGE and immunoblotted using a MAb specific for μ1. The position of the δ band is shown. (E) ISVPs of rsT3D or the indicated μ1 mutant were incubated at the temperatures shown for 15 min. Residual infectivity was assessed by plaque assay. Results are expressed as mean residual titer for triplicate samples. Error bars indicate SD.

The μ1 ϕ domain incorporates an amphipathic helix that is hypothesized to function during membrane penetration [24],[36]. To determine whether mutations in ϕ alter reovirus membrane-penetration efficiency, we tested the capacity of ISVPs generated from each mutant virus to lyse erythrocytes (Figure 3A). The property of reovirus to perturb erythrocyte membrane integrity and cause hemolysis correlates with endosomal membrane penetration [12],[29],[31]. Incubation of bovine erythrocytes with either rsT3D or I595K resulted in efficient erythrocyte lysis, whereas the K594D mutant failed to elicit significant levels of hemolysis. To determine whether the K594D mutant also is altered in penetration of membranes of reovirus-permissive cells, we tested the capacity of wild-type and ϕ mutant viruses to mediate the release of ^51^Cr from preloaded L929 cells (Figure 3B). Incubation of L929 cells with either rsT3D or I595K resulted in significantly greater ^51^Cr release than did K594D. These data suggest an important role for ϕ residue Lys594 in membrane penetration.

As a surrogate measure of the capacity of reovirus to deliver its core into the cytoplasm, we tested the capacity of rsT3D and the ϕ mutants to allow α-sarcin coentry as a consequence of reovirus-mediated membrane breach (Figure 3C). Inhibition of cellular protein synthesis following coentry of α-sarcin is determinative of efficient membrane penetration by reovirus [12],[29],[37]. Quantification of protein-synthesis inhibition 1 h following infection revealed that rsT3D and I595K efficiently inhibited protein synthesis in the presence of α-sarcin, whereas K594D did not. Consistent with results from hemolysis and chromium-release assays, these findings indicate that ϕ residue Lys594 regulates viral membrane-penetration efficiency.

### Mutations in ϕ affect ISVP* formation

Formation of size-selective pores in host cell membranes is thought to be an essential event during membrane penetration by reovirus [31]. Conformational changes in μ1 that accompany the conversion of ISVPs to ISVP*s are prerequisite for formation of pores [28],[29],[31]. To determine whether mutations in ϕ diminish membrane-penetration efficiency by preventing these conformational changes, we evaluated the capacity of K594D and I595K to form ISVP*s in vitro. As a control, we used a membrane penetration-defective δ mutant virus, A319E [12],[34],[38], which is inefficient in ISVP* formation [29]. ISVPs of wild-type and each mutant virus were incubated with Cs ions, which favor ISVP* formation [28] and probed for the susceptibility of the μ1 δ fragment to trypsin digestion by immunoblotting (Figure 3D). Increased susceptibility of the ISVP-associated δ fragment to trypsin in the presence of Cs correlates with ISVP* formation [29]. Trypsin treatment of Cs-treated ISVPs of either rsT3D or I595K resulted in rapid degradation of the μ1 δ fragment. In contrast, the δ fragments of Cs-treated ISVPs of both K594D and A319E remained resistant to trypsin cleavage for an extended interval, with the A319E δ fragment displaying greater trypsin resistance than the δ fragment of K594D. These data indicate that ISVP-to-ISVP* conversion is affected by the K594D mutation but not the I595K mutation.

As an additional measure of the diminished capacity of μ1 to undergo conformational changes consistent with ISVP* formation, we evaluated residual infectivity of reovirus ISVPs following exposure to increasing temperature (Figure 3E). Reduction in the efficiency of ISVP-to-ISVP* transition correlates with enhanced thermal stability of reovirus ISVPs [39]. Incubation of either rsT3D or I595K ISVPs for 15 min at temperatures≥42°C resulted in at least a 10 000-fold reduction in infectivity. In contrast, infectivity of A319E ISVPs remained unchanged at temperatures up to 47°C and was diminished only ∼100-fold following incubation at 52°C. Like A319E, incubation of K594D ISVPs at temperatures up to 42°C had no effect on infectivity. However, ∼100- and 10 000-fold decreases in K594D infectivity were observed following incubation at 47°C and 52°C, respectively. These data demonstrate that K594D displays thermal stability intermediate to that of rsT3D and A319E and suggest that Lys594 modulates the conformational flexibility of μ1.

### Reovirus ϕ mutants are apoptosis defective

**Figure 4:**
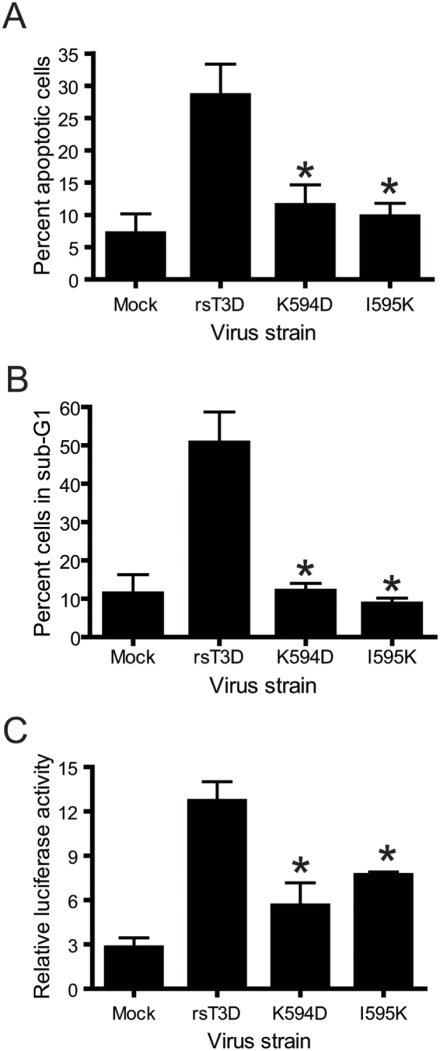
K594D and I595K are apoptosis-defective. (A) HeLa cells were infected with rsT3D or the indicated ϕ mutant at an MOI of 100 PFU/cell. Following 48 h incubation, the percentage of apoptotic cells was determined after staining with acridine orange. Results are expressed as the mean percentage of apoptotic cells for triplicate samples. Error bars indicate SD. *, *P*<0.05 as determined by Student's *t*-test in comparison to rsT3D. (B) HeLa cells were infected with rsT3D or the indicated ϕ mutant at an MOI of 100 PFU/cell. Following 48 h incubation, the percentage of cells with less than G1 DNA content (sub-G1) was quantified by staining with propidium iodide. Results are expressed as the mean percentage of sub-G1 cells for triplicate samples. Error bars indicate SD. *, *P*<0.05 as determined by Student's *t*-test in comparison to rsT3D. (C) 293T cells were co-transfected with pNF-κB-Luc and pRenilla-Luc 24 h prior to adsorption with rsT3D or the indicated ϕ mutant at an MOI of 100 PFU/cell. Luciferase activity in cell lysates was determined at 24 h post-infection. Results are presented as the ratio of normalized luciferase activity from infected cell lysates to that from mock-infected lysates for triplicate samples. Error bars indicate SD. *, *P*<0.05 as determined by Student's *t*-test in comparison to rsT3D.

Residues Lys594 and Ile595 in μ1 ϕ modulate apoptosis induction following ectopic expression in cells (Figure 1). To determine whether these residues also are important for apoptosis induction during reovirus infection, we compared the apoptotic potential of wild-type and ϕ mutant viruses by acridine orange staining (Figure 4A). Following infection of HeLa cells with rsT3D, ∼30–35% of cells showed altered nuclear morphology characteristic of apoptosis. However, infection with the K594D or I595K elicited apoptosis in only ∼12% of cells. As a complementary approach, we used propidium iodide staining and flow cytometry to compare the capacity of these viruses to promote DNA fragmentation in infected cells (Figure 4B). Infection of HeLa cells with rsT3D resulted in ∼50% cells displaying hypodiploid (sub-G1) DNA staining, characteristic of apoptotic cells. In contrast, infection with either K594D or I595K resulted in a percentage of cells in sub-G1 that was comparable to mock infection. These results suggest that the apoptotic potential of reovirus is regulated by ϕ residues Lys594 and Ile595.

Activation of transcription factor NF-κB is required for induction of apoptosis in cultured cells following infection with reovirus [40],[41]. To test whether the limited apoptotic potential of the ϕ mutants is linked to a defect in the activation of NF-κB signaling, the capacity of wild-type and mutant viruses to stimulate NF-κB activity was quantified using a reporter assay (Figure 4C). For these experiments, 293T cells were transfected with a reporter plasmid that expresses firefly luciferase under the control of an NF-κB-regulated promoter. A plasmid that constitutively expresses Renilla luciferase was used to normalize for transfection efficiency. In comparison to mock-infected 293T cells, infection with rsT3D resulted in ∼4-fold increase in NF-κB-dependent gene expression. In contrast, no more than 2.5-fold stimulation of NF-κB reporter gene activity was observed following infection with the ϕ mutants K594D or I595K. These data suggest that the residues in ϕ required for apoptosis induction also regulate NF-κB activation following reovirus infection. Furthermore, since the I595K mutant effectively penetrates membranes but fails to efficiently activate proapoptotic signaling, these findings indicate that the membrane-penetration and apoptosis-induction functions of the μ1 ϕ domain can be genetically uncoupled. Collectively, these results suggest that initiation of reovirus-induced proapoptotic signaling is mediated by ϕ and occurs following penetration of endosomal membranes.

### ϕ mutant μ1 associates inefficiently with intracellular membranes

**Figure 5:**
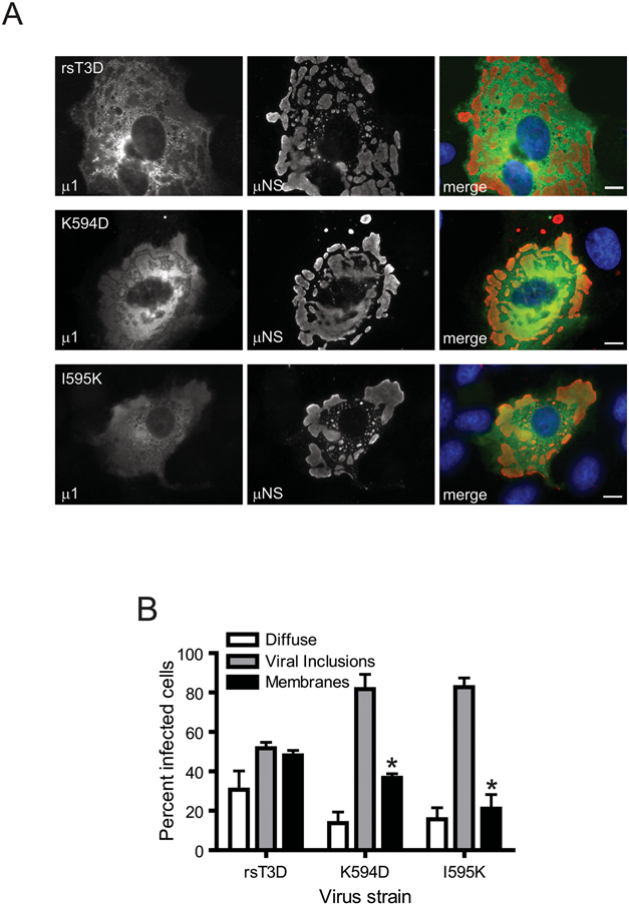
Apoptosis-modulating ϕ mutations alter μ1 distribution in cells. (A) CV-1 cells were infected with 10 PFU/cell of rsT3D or the indicated ϕ mutant, fixed 48 h post-infection, permeabilized, and immunostained with anti-μ1 MAb 4A3 (green) and anti-μNS serum (red), followed by goat anti-mouse IgG conjugated to Alexa Fluor 488 (green) and goat anti-rabbit IgG conjugated to Alexa Fluor 594 (red). Scale bars, 10 µm. A representative image of the predominant μ1 distribution pattern following infection with each virus strain is shown. (B) The patterns of μ1 distribution in individual infected cells were scored for each time point as diffuse, associated with viral inclusions, or associated with intracellular membranes (marked by distinct ring-like distribution of μ1). Results are expressed as the mean percentage of cells showing the indicated μ1 distribution for triplicate samples. Error bars indicate SD. *, *P*<0.05 as determined by Student's *t*-test in comparison to rsT3D.

The capacity of ectopically expressed μ1 to induce apoptosis correlates with its distribution to intracellular membranous structures such as mitochondria, endoplasmic reticulum, and lipid droplets [33]. Moreover, a short loop and amphipathic helix within φ (residues 582 to 611) is necessary and sufficient for association of μ1 with intracellular membranous structures and induction of apoptosis [33] and contains residues Lys594 and Ile595. To determine whether the decrease in apoptotic potential of ϕ mutants K594D and I595K resulted from a diminished capacity of mutant μ1 to associate with intracellular membranes, we compared the distribution of wild-type and mutant μ1 proteins in infected CV-1 cells by immunofluorescence microscopy (Figure 5A). Consistent with previous findings [33], infection with rsT3D and each of the ϕ mutant viruses resulted in three discernable μ1 distribution patterns: (i) diffuse cytoplasmic distribution, (ii) incorporation within viral inclusions, or (iii) localization to intracellular membranes structures. Although μ1 was found in all three locations following infection with rsT3D, K594D, and I595K, a lower fraction of cells displayed diffuse μ1 localization following infection with either of the ϕ mutants (∼15%) in comparison to rsT3D (∼30%) (Figure 5B). Similarly, μ1 localized to intracellular membrane fractions in a significantly lower percentage of cells following infection with either K594D (∼35%) or I595K (∼20%) in comparison to rsT3D (∼50%). Concordant with the decrease in μ1 distribution throughout the cytoplasm and to intracellular membranes, a considerably higher fraction of cells showed μ1 within viral inclusions following infection with the ϕ mutants (∼80%) in comparison to wild-type virus (∼50%). Thus, mutations in ϕ that modulate apoptosis induction affect μ1 distribution in cells.

### Mutations in ϕ diminish reovirus virulence in the CNS

**Figure 6:**
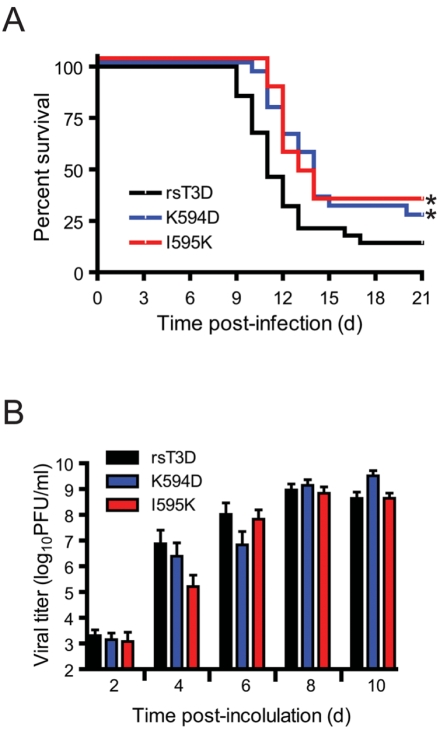
Apoptosis-defective ϕ mutants display attenuated virulence. ND4 Swiss Webster mice were inoculated intracranially with 50 PFU of rsT3D or the indicated ϕ mutant. (A) Mice (18–20) were monitored for survival over 21 days. *, *P*<0.05 as determined by logrank test in comparison to rsT3D. (B) Brains from infected mice were resected at the times shown and homogenized by freeze-thaw and sonication. Viral titers in brain homogenates were determined by plaque assay. Results are expressed as the mean viral titer (for 6–12 animals). Error bars indicate SD.

Intracranial inoculation of 2 d old mice with type 3 reovirus strains such as T3D at doses as low as 10 PFU produces lethal encephalitis [7]–[9]. To determine whether in vitro differences in the apoptosis-inducing capacity of ϕ mutants affect reovirus disease, we inoculated newborn mice intracranially with 50 PFU of either rsT3D or the apoptosis-defective ϕ mutants, K594D or I595K, and monitored mice for survival and signs of neurological disease for an interval of 21 d (Figure 6A). We noted a significant difference in the survival of mice infected with rsT3D and either K594D or I595K. The median survival for rsT3D-infected mice was 11 d. In contrast, median survival for K594- or I595K-infected mice was 13 d. Moreover, a substantially greater proportion of mice survived infection with the ϕ mutant viruses (∼30%) in comparison to those infected with rsT3D (∼15%). These data indicate that ϕ residues Lys594 and Ile595 influence reovirus neurovirulence.

To determine whether the delay in disease onset following infection with the ϕ mutants results from delayed viral replication kinetics, mice infected with rsT3D, K594D, and I595K were euthanized at 2, 4, 6, 8, and 10 d after infection, and viral titers in resected brains were quantified by plaque assay (Figure 6B). Following infection with rsT3D, viral titers increased rapidly in the mouse brain to reach a peak titer of ∼10^9^ PFU/ml by 8 d post-inoculation. Apoptosis-defective mutants K594D and I595K also displayed similar replication kinetics and attained titers equivalent to rsT3D by 8 d post-inoculation. These data indicate that K594D and I595K replicate as efficiently as wild-type virus in the murine CNS and that alterations in viral replication kinetics in vivo are not likely to contribute to the decreased capacity of ϕ mutant viruses to produce fatal encephalitis.

**Figure 7:**
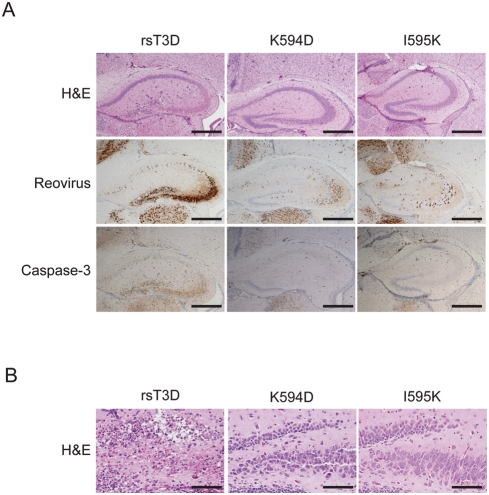
ϕ mutant viruses produce less histopathologic injury than wild-type virus. ND4 Swiss Webster mice were inoculated intracranially with 50 PFU of rsT3D or the indicated ϕ mutant. (A) Brains from infected mice were resected at 10 d post-inoculation, fixed, and embedded in paraffin. Brain sections were stained with H&E, polyclonal reovirus-specific antiserum, or activated caspase-3-specific antiserum. Consecutive sections from the hippocampal region of representative brains of rsT3D-, K594D-, or I595K-infected mice are shown. Scale bars, 500 µm. (B) Higher magnification images of H&E-stained sections of the dentate gyrus and CA4 region of the hippocampus of mice infected with each virus strain are shown. Scale bars, 100 µm.

To evaluate the extent of injury in the CNS of mice infected with rsT3D, K594D, and I595K, we examined hematoxylin and eosin (H&E)-stained, coronal brain sections from mice euthanized at 10 d following intracranial viral inoculation (Figure 7A, top panel and 7B). Mice infected for 10 d were selected for these histopathological analyses since virus strains used in this study displayed maximal titers at that time point. Sections from rsT3D-infected mice showed the presence of reactive blood vessels and infiltration by inflammatory cells and extensive neuronal death in the cerebral cortex, hippocampus, thalamus, and hypothalamus. Consistent with previous reports [10]–[12], these characteristic pathological features indicate that intracranial inoculation of rsT3D results in overt meningoencephalitis. The hippocampal region of mice infected with rsT3D showed extensive damage to layers CA3 and CA4, with the pyramidal cells displaying classical apoptotic nuclear morphology (Figure 7B). In addition, microcystic changes and neuronal apoptosis also were evident in the dentate gyrus of rsT3D-infected mice. In contrast, the hippocampal region of the brains of mice inoculated with either K594D or I595K displayed minimal damage, with evidence of only focal apoptosis or necrosis in both the CA3 layer and the dentate gyrus (Figure 7B). These findings suggest that K594D and I595K produce less pathological injury than wild-type virus in the murine CNS.

To determine whether the observed differences in severity of injury result from alterations in viral tropism due to mutations at Lys594 and Ile595, infected mouse brain sections consecutive to those used in the H&E analyses were stained with reovirus-specific antiserum (Figure 7A, middle panel). Immunoreactive neurons were detected in the cerebral cortex, hippocampus, thalamus, and hypothalamus of mice infected with rsT3D, K594D, and I595K. Despite discernable differences in virus-induced cell death and inflammation in mouse brains infected with rsT3D and ϕ mutant viruses, localization of viral antigen was similar in mice infected with both types of viruses. Although differences are evident in the intensity of reovirus-specific staining in the hippocampus of mice infected with rsT3D, K594D, or I595K, the specific brain regions infected by these viruses were similar (Figure 6B). These data indicate that mutations in ϕ that affect either its membrane-penetration or apoptosis-induction function do not alter reovirus tropism.

To qualitatively assess whether rsT3D and the ϕ mutants vary in the capacity to elicit apoptosis in vivo, sections from infected mouse brains were stained with an antiserum specific for the activated form of caspase-3 as a biochemical marker for apoptosis (Figure 7A, lower panel). In mice infected with rsT3D, regions of the hippocampus immunoreactive for viral antigen also were positive for activated caspase-3. In contrast, a substantially lower fraction of cells stained for activated caspase-3 following infection with either K594D or I595K. These data suggest that the diminished virulence of K594D and I595K is a consequence of decreased apoptotic potential.

### ϕ mutants are apoptosis-defective in vivo

**Figure 8:**
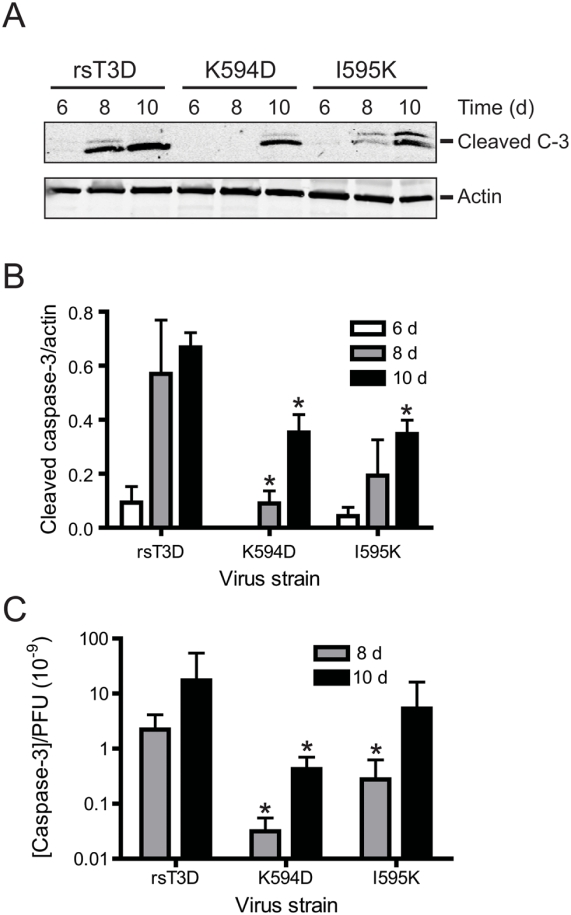
ϕ mutant viruses are apoptosis-defective in vivo. ND4 Swiss Webster mice were inoculated intracranially with 50 PFU of rsT3D or the indicated ϕ mutant. Brains from infected mice were resected at the times shown and homogenized by freeze-thaw and sonication. (A) Brain homogenates were resolved by SDS-PAGE and immunoblotted using an antiserum specific for activated caspase-3 (C-3) (upper panel). As a control for protein concentration, the blots were stripped and reprobed with an antibody specific for actin (lower panel). (B) Brain homogenates from five animals infected with each virus strain for the times shown were resolved by SDS-PAGE and immunoblotted using antisera specific for either activated caspase-3 or actin. Pixel intensities of activated caspase-3 and actin bands were quantified by densitometry. Results are expressed as the mean ratio of activated caspase-3 band intensity to that of actin. Error bars indicate SEM. *, *P*<0.05 as determined by Student's *t* test in comparison to rsT3D at each time point. (C) For each brain homogenate, the cleaved caspase-3/actin ratio obtained by immunoblot analysis was divided by the virus titer. Results are expressed as the mean activated caspase-3 concentration/PFU. Error bars indicate SEM. *, *P*<0.05 as determined by Student's *t* test in comparison to rsT3D at each time point.

To quantitatively test whether the ϕ mutant viruses differ from wild-type virus in apoptosis-inducing capacity, we examined brain homogenates at 6, 8, and 10 d post-inoculation for the activated form of caspase-3 by immunoblotting (Figure 8A). Activated caspase-3 levels in the brain homogenates of five randomly selected mice following infection with each virus strain were normalized to the levels of actin (Figure 8B). At each interval tested, brain homogenates from mice infected with rsT3D displayed a substantially higher level of activated caspase-3 in comparison to those infected with either K594D or I595K. Although the mean viral titers in the brains of mice infected with rsT3D, K594D, and I595K are equivalent (Figure 6B), it is possible that differences in the concentration of activated caspase-3 in an individual mouse brain (shown in Figure 7B) are a consequence of differences in viral replication. To control for such differences, the in vivo apoptotic potential of each virus was expressed as a ratio of the concentration of activated caspase-3 per PFU of virus (Figure 8C) for each infected mouse brain. In comparison to rsT3D, we found significantly lower levels of activated caspase-3/PFU following infection with K594D (∼70-fold) and I595K (∼10-fold) at 8 d post infection. Although apoptosis levels increase at 10 d post infection following infection with each virus, the activated caspase-3/PFU ratio remained lower following infection with K594D (∼8-fold) and I595K (∼3-fold) in comparison to rsT3D. These data indicate that the ϕ mutant viruses are defective in apoptosis induction in vivo and provide further evidence that the attenuated virulence of K594D and I595K is attributable to a reduced capacity to evoke apoptotic cell death.

## Discussion

Apoptosis induction following reovirus infection is regulated by the μ1 viral outer-capsid protein. In this study, we evaluated the role of the μ1 ϕ domain in apoptosis induction following reovirus infection by characterizing viruses with single amino acid substitutions within ϕ. We found that the reovirus ϕ domain regulates two important processes in the reovirus infectious cycle. First, residues in ϕ influence reovirus membrane-penetration efficiency during cell entry by preventing conformational changes in μ1 that accompany the ISVP-to-ISVP* transition. Second, mutations in ϕ affect the capacity of reovirus to activate NF-κB and induce apoptosis. Importantly, these effects are independent: I595K, an apoptosis-defective ϕ mutant, is fully capable of penetrating membranes. These findings highlight a critical function for the μ1 ϕ domain in both membrane penetration and apoptosis induction following reovirus infection and suggest that initiation of apoptosis following reovirus infection occurs subsequent to penetration of endosomal membranes.

Structure-function relationships in μ1 that govern its membrane-penetration activity are poorly defined. Following generation of ISVPs, conformational changes in μ1 triggered by the endocytic milieu lead to generation of ISVP*s [28]. These conformational changes facilitate membrane penetration by promoting efficient cleavage and release of the myristoylated N-terminal μ1 fragment, μ1N [30],[42], which interacts with membranes to allow osmotic rupture [31],[32]. Analogous to μ1N, the ϕ fragment also is released from the particle during ISVP-to-ISVP* transition triggered by exposure to CsCl and associates with target membranes [32]. Although released ϕ is not sufficient for membrane penetration, μ1N-mediated membrane penetration is more efficient in the presence of released ϕ [32]. Based on these observations, ϕ has been hypothesized to function as a μ1N chaperone and facilitate membrane penetration by enhancing μ1N solubility or preventing nonproductive interactions between μ1N molecules [32]. Results presented here suggest that ϕ residue Lys594 regulates conformational changes that accompany ISVP-to-ISVP* formation. Since release of the myristoylated μ1N fragment requires ISVP* formation, the diminished membrane-penetration efficiency of K594D is likely attributable to the decreased propensity of the mutant μ1 protein to undergo the conformational changes that lead to release of μ1N. Although our data do not exclude a role for ϕ as a μ1N chaperone, results reported here indicate an additional mechanism by which ϕ influences μ1N function.

Analysis of the ISVP*-associated μ1 protein using antibodies that recognize conformation-specific epitopes within δ and measurement of δ protease sensitivity indicate that ISVP* formation is accompanied by changes in δ conformation [29]. Concordantly, amino acid substitutions in δ that enhance thermal stability of reovirus particles [39] or render them resistant to 33% ethanol [34],[38] prevent conformational changes in δ. Data gathered in this study indicate that mutations within ϕ also modulate conformational rearrangements in δ during ISVP* formation. Since both μ1N and ϕ are released from the virus particle during ISVP* formation [32], it is likely that conformational rearrangements in μ1 during ISVP* formation are not restricted to the δ domain and also involve the μ1N and ϕ domains. The model of membrane penetration suggested by the crystal structure of the native μ1 trimer also supports the idea that a coordinated set of conformational rearrangements are required to allow release of these cleavage fragments [36],[43]. Therefore, amino acid substitutions within ϕ that negatively affect the conformational flexibility of ϕ would likely prevent μ1 reorganization required for ISVP* formation. Biochemical and structural characterization of additional mutant viruses that may be affected to varying degrees in the capacity to undergo μ1 conformational changes may identify as yet unknown intermediates during ISVP-to-ISVP* conversion and offer insight into mechanisms that promote the elaborate remodeling of the μ1 protein that is required to mediate membrane penetration.

Correlation of reovirus membrane-penetration efficiency with its apoptotic potential suggests that the apoptotic cascade is initiated as a consequence of membrane penetration [12]. However, prior to this study, it was not apparent whether the linkage between membrane penetration and apoptosis induction was direct or indirect. Findings presented here demonstrate that a membrane penetration-proficient μ1 mutant is apoptosis-defective, indicating that the ϕ domain activates prodeath signaling subsequent to membrane penetration. It is probable that like the δ domain [29], the released ϕ domain also gains access to the cytoplasm after penetration of endosomal membranes. We hypothesize that following delivery into the cytoplasm, the ϕ domain engages a cellular sensor to activate NF-κB and possibly other proapoptotic signaling pathways that function cooperatively to evoke cell death [44]–[46]. Although the fate of released ϕ following successful membrane penetration during reovirus entry is unknown, ϕ may localize to intracellular membranous structures such as mitochondria, ER, and lipid droplets similar to that observed following ectopic expression of epitope-tagged ϕ in transfected cells [33]. Since localization of μ1 to these sites is required for its prodeath function and is attributable to residues 582–611 within the ϕ fragment [33], distribution of de novo synthesized μ1 was used as a surrogate for ϕ distribution following cell entry of reovirus. Consistent with previous observations, the K594D and I595K μ1 proteins displayed subcellular distribution that differed from wild-type μ1. In comparison with the wild-type μ1 protein, the mutant μ1 proteins were found to localize less efficiently to intracellular membranes. In addition, ϕ mutant μ1 proteins also displayed diminished localization to the cytoplasm. These findings are consistent with the idea that ϕ initiates prodeath signaling following localization to intracellular membranes or cytoplasm.

We envision three mutually non-exclusive mechanisms by which ϕ may modulate prodeath signaling. First, ϕ may activate apoptotic signaling via engagement of a cell-death regulator resident either in the mitochondria or ER. For example, ϕ could interact with and modify the function of either the proapoptotic or anti-apoptotic members of the Bcl-2 family that are known to localize to both of these organelles [47]. These interactions could directly activate classical apoptotic pathways that contribute to reovirus-induced cell death. Second, ϕ could stimulate prodeath signaling by affecting mitochondrial membrane potential [48],[49] or calcium ion levels in the ER lumen [50]. In support of this possibility, ϕ preferentially localizes to these organelles [33] and contains an amphipathic helix [24],[36] that may be capable of pore formation following interaction with membranes. Third, ϕ might activate NF-κB through engagement of a cytoplasmic or organelle-associated cellular factor that stimulates IκB kinase (IKK) activity. Although it is known that reovirus-induced NF-κB activation requires the function of IκB kinase (IKK) complex components IKKα and IKKγ/NEMO [41], the signal transducer that couples reovirus to these IKK subunits is not defined. ϕ also may activate IKKs via direct interaction with IKK components analogous to the HTLV Tax protein, which stimulates IKK activity via interaction with IKKγ/NEMO [51],[52]. Our future studies will elucidate how ϕ evokes proapoptotic signaling.

In addition to enhancing an understanding of molecular mechanisms underlying reovirus-induced proapoptotic signaling, our findings also indicate that the prodeath function of ϕ modulates reovirus neurovirulence. These findings are consistent with previous studies that link the apoptosis-inducing capacity of reovirus to its virulence potential [10]–[14] and suggest that apoptosis efficiency controls virulence through adverse effects on viral replication or by interfering with host physiology. Since diminishment in apoptotic efficiency of ϕ mutant reoviruses is not associated with a reduction in viral replication efficiency at the target site of disease, our data suggest that apoptosis directly affects reovirus pathogenesis. Because infection by type 3 reoviruses such as those used in this study leads to encephalitis marked by destruction of a non-renewable, post-mitotic neuronal cell population, it is possible that the morbidity and mortality caused by reovirus infection in mice is a consequence of apoptotic cell death of neurons that control essential host functions. Our findings also indicate that in comparison to the ϕ mutant viruses, infection by wild-type rsT3D results in greater influx of inflammatory cells, suggesting that inflammation-induced injury to neurons contributes to reovirus pathogenesis. This conclusion is consistent with previously suggested roles for inflammatory mediators such as IL-1β in reovirus CNS disease [53]. Although it is not known how inflammation is initiated in response to viral infection, it is possible that reovirus-induced activation of NF-κB, which is a known proinflammatory transcription factor [54], also contributes to viral disease independent of effects on virus-induced apoptosis.

Analogous to reovirus, apoptosis induction by dengue virus [2], rabies virus [55], Sindbis virus [56], and West Nile virus [57] correlate with neurovirulence potential. Since viral encephalitis appears to be a common consequence of neuronal apoptosis, these findings collectively identify virus-induced proapoptotic signaling as a viable target for therapy against viruses that infect the CNS. Consistent with this relationship, agents that protect neurons from apoptotic cell death ameliorate viral encephalitis [13],[14],[57]. Results presented here enhance an understanding of events at the virus-host interface that lead to activation of prodeath signaling and provide a platform for illumination of therapeutic targets that may be broadly applicable to encephalitic viruses.

## Materials and Methods

### Cells

HeLa cells and 293T cells were maintained in DMEM (Invitrogen) supplemented to contain 10% fetal bovine serum (FBS), 2 mM L-glutamine, 100 U/ml of penicillin, 100 µg/ml streptomycin, and 25 ng/ml of amphotericin B (Sigma-Aldrich). L929 cells were maintained in Joklik's MEM (Sigma-Aldrich) supplemented to contain 10% FBS, 2 mM L-glutamine, 100 U/ml of penicillin, 100 µg/ml streptomycin, and 25 ng/ml of amphotericin B. CV-1 cells were maintained in Ham's F-12 medium (CellGro) supplemented to contain 10% FBS, 100 U/ml penicillin, 100 µg/ml streptomycin, 1 mM sodium pyruvate, and nonessential amino acids (CellGro).

### Viruses

Recombinant reoviruses were generated by plasmid-based reverse genetics [35]. Monolayers of L929 cells at approximately 90% confluence (3×10^6^ cells) in 60 mm dishes (Costar) were infected with rDIs-T7pol [58] at an MOI of ∼ 0.5 TCID_50_. At 1 h post-infection, cells were cotransfected with ten plasmid constructs representing the cloned T3D genome using 3 µl of TransIT-LT1 transfection reagent (Mirus) per µg of plasmid DNA. Following 5 d of incubation, recombinant virus was isolated from transfected cells by plaque purification using monolayers of L929 cells [59]. For generation of μ1 mutant viruses, pT7-M2T3D [35] was altered by Quickchange (Stratagene) site-directed mutagenesis. To confirm sequences of mutant viruses, viral RNA was extracted from purified virions and subjected to Onestep RT-PCR (Qiagen) using L1 or M2-specific primers. (Primer sequences are available from the corresponding authors upon request.) The purified PCR products were subjected to sequence analysis for the presence of the introduced mutation in the M2 gene segment and the noncoding signature mutation in the L1 gene segment [35].

Purified reovirus virions were prepared using second- or third-passage L929-cell lysate stocks of twice plaque-purified reovirus as described [60]. Viral particles were Freon-extracted from infected cell lysates, layered onto CsCl gradients, and centrifuged at 62,000×*g* for 18 h. Bands corresponding to virions (1.36 g/cm^3^) [61] were collected and dialyzed in virion-storage buffer (150 mM NaCl, 15 mM MgCl_2_, 10 mM Tris-HCl [pH 7.4]). The concentration of reovirus virions in purified preparations was determined from an equivalence of one OD unit at 260 nm equals 2.1×10^12^ virions [61]. Viral titer was determined by plaque assay using L929 cells [59]. ISVPs were generated by incubation of 2×10^12^ virions with 200 µg/ml of *N*-*p*-tosyl-L-lysine chloromethyl ketone-treated chymotrypsin in a total volume of 0.1 ml at 37°C for either 20 min (for Cr- release assays) [34] or 1 h [62]. Proteolysis was terminated by addition of 2 mM phenylmethylsulphonyl fluoride and incubation of reactions on ice. Generation of ISVPs was confirmed by SDS-polyacrylamide gel electrophoresis and Coomassie Brilliant Blue staining.

### Antibodies and plasmids

Antisera raised against T1L and T3D [63], μNS [64], and μ1-specific monoclonal antibody (MAb) 4A3 [65] have been described. Rabbit antiserum specific for activated caspase-3 was purchased from Cell Signaling. GFP-specific MAb was purchased from Clontech. Goat antiserum specific for actin was purchased from Santa Cruz Biotechnology. Mouse ascites specific for the PSTAIR peptide of Cdk1 was purchased from Sigma-Aldrich. Alexa Fluor-conjugated anti-mouse IgG, anti-rabbit IgG, and anti-goat IgG secondary antibodies were purchased from Invitrogen.

The μ1 ORF was amplified by PCR from viral RNA and cloned into pCR-script (Stratagene). The *XhoI*-*NotI* fragment released from pCR-script was ligated into complementary restriction sites in pCI-neo (Promega) to generate pCI-M2. Plasmids encoding mutant μ1 proteins were generated using pCI-M2 and Quickchange site-directed mutagenesis. The T3D ϕ ORF was amplified by PCR from pT7-M2T3D and cloned into pCR-script. The XhoI-SmaI fragment released from pCR-script was ligated into complementary restriction sites in pEGFP-C1 (Clontech) to generate GFP-tagged ϕ.

Plasmids pRenilla-Luc and pNF-κB-Luc [51] were obtained from Dr. Dean Ballard.

### Assessment of μ1 and GFP-ϕ expression by immunoblotting

293T cells (2×10^5^) in 24-well plates (Costar) were treated with 50 µM Z-VAD-FMK (Biomol) and transfected with 0.8 µg/well of plasmids expressing wild-type or mutant T3D μ1 or GFP-ϕ proteins using Lipofectamine 2000 (Invitrogen) according to the manufacturer's instructions. At 24 h following transfection, cells were washed in phosphate-buffered saline (PBS) and lysed with 1× RIPA (50 mM Tris [pH 7.5], 50 mM NaCl, 1% TX-100, 1% DOC, 0.1% SDS, and 1 mM EDTA) containing a protease inhibitor cocktail (Roche). Following centrifugation at 15 000×*g* to remove debris, the cell lysates were resolved by electrophoresis in 4–20% polyacrylamide gels and transferred to nitrocellulose membranes. Membranes were blocked for at least 1 h in blocking buffer (PBS containing 5% milk) and incubated with 1 µg/ml of anti-μ1 MAb 4A3 or an anti-GFP MAb and anti-PSTAIR ascites diluted 1∶10 000 in blocking buffer at room temperature for 1 h. Membranes were washed three times for 10 min each with washing buffer (PBS containing 0.1% Tween-20) and incubated with Alexa Fluor 680-conjugated goat anti-mouse Ig diluted 1∶10 000 in blocking buffer. Membranes were washed three times and scanned using a Odyssey Infrared Imager (LiCor).

### Assessment of caspase-3/7 activity

293T cells (2×10^5^) in 24-well plates were transfected with 0.8 µg/well of plasmids expressing wild-type or mutant μ1 GFP-ϕ proteins using Lipofectamine 2000 according to the manufacturer's instructions. Plates were frozen 24 h post-transfection, and caspase-3/7 activity in thawed lysates containing 5×10^3^ cell equivalents was quantified using the Caspase-Glo-3/7 assay system (Promega) according to the manufacturer's instructions.

### Assessment of viral infectivity by indirect immunofluorescence

L929 cells (2×10^5^) in 24-well plates were adsorbed with reovirus at room temperature for 1 h. Following removal of the inoculum, cells were washed with PBS and incubated in complete medium at 37°C for 18 h to permit completion of a single cycle of viral replication. Monolayers were fixed with methanol, washed twice with PBS, blocked with 2.5% immunoglobulin-free bovine serum albumin (Sigma-Aldrich) in PBS, and incubated successively for 1 h with polyclonal rabbit anti-reovirus serum at a 1∶1000 dilution and a 1∶1000 dilution of Alexa Fluor 546-labeled anti-rabbit IgG. Monolayers were washed with PBS, and infected cells were visualized by indirect immunofluorescence using a Zeiss Axiovert 200 fluorescence microscope. Infected cells were identified by the presence of intense cytoplasmic fluorescence that was excluded from the nucleus. No background staining of uninfected control monolayers was observed. Reovirus antigen-positive cells were quantified by counting fluorescent cells in at least two random fields of view in triplicate wells at a magnification of 20× or by counting the entire well for duplicate wells.

### Assessment of virus replication by plaque assay

L929 cells (2×10^5^) in 24-well plates were adsorbed in triplicate with reovirus at room temperature for 1 h in serum-free medium, washed once with PBS, and incubated in serum-containing medium for various intervals. Cells were frozen and thawed twice prior to determination of viral titer by plaque assay using L929 cells [59]. Viral yields were calculated according to the following formula: log_10_yield_x_ = log_10_(PFU/ml)_x_−log_10_(PFU/ml)_0_, where x is the time post-infection.

### Hemolysis assay

Citrated calf red blood cells (RBCs) (Colorado Serum Company) were washed extensively with chilled PBS supplemented to contain 2 mM MgCl_2_ (PBS-Mg) and resuspended at a concentration of 30% v/v in PBS-Mg. Hemolysis efficiency was analyzed by mixing a 3 µl aliquot of resuspended RBCs with virion-storage buffer, 1% TX-100 in virion-storage buffer, or ISVPs in a total volume of 30 µl, followed by incubation at 37°C for 1 h. Samples were placed on ice for 30 min to prevent further hemolysis and centrifuged at 500×*g* at 4°C for 3 min. The extent of hemoglobin release was quantified by measuring the A_415_ of a 1∶5 dilution of the supernatant in a microplate reader (Molecular Devices). Percentage hemolysis was calculated using the following formula: % hemolysis = 100×(A_sample_−A_buffer_)/(A_TX-100_−A_buffer_).

### Chromium-release assay

A 0.5 ml suspension of L929 cells at a concentration of 4×10^6^ cells/ml was incubated in serum-free medium supplemented to contain 100 µCi ^51^Cr-labeled sodium chromate (Perkin Elmer) at 37°C for 2 h. Cells were pelleted by centrifugation at 1000×*g* for 5 min, washed twice with chilled PBS, and divided into aliquots of 6×10^4^ cells. Cells were adsorbed with ISVPs of each virus strain at 4°C for 1 h. Infection was initiated following removal of unbound ISVPs by washing with chilled PBS and addition of 0.25 ml pre-warmed complete medium. Spontaneous and total ^51^Cr release from cells was determined following mock infection or treatment with 4% Triton X-100, respectively. Cells were transferred to 96-well plates (Costar) and incubated at 37°C for 4 h. The plates were centrifuged at 1000×g at room temperature for 5 min to pellet unattached cells. The amount of ^51^Cr released into 25 µl of medium was quantified by liquid scintillation. Percent lysis was calculated using the following formula: % lysis = 100×(cpm_sample_−cpm_mock_)/(cpm_TX-100_−cpm_mock_).

### α-sarcin coentry assay

HeLa cells (2×10^4^) in 96-well plates were preincubated in serum-free, cysteine- and methionine-free DMEM (Invitrogen) at 37°C for 3 h. Cells were pre-chilled at 4°C for 1 h and adsorbed with ISVPs at 4°C for 1 h. Infection was initiated following removal of unbound ISVPs by washing with chilled PBS and addition of pre-warmed, cysteine- and methionine-free DMEM supplemented to contain 5% FBS, 22 µCi/ml of ^35^S-labeled methionine-cysteine (Perkin Elmer), and either 0 or 50 µg/ml α-sarcin (Calbiochem). After incubation at 37°C for 1 h, cells were lysed using 1× RIPA buffer, and proteins were precipitated following addition of chilled trichloroacetic acid (TCA) to a final concentration of 30% and incubation overnight at 4°C. Precipitates were washed successively with 10% TCA and 95% ethanol, air dried, and solubilized in 50 mM unbuffered Tris containing 0.05% SDS. TCA-precipitable radioactive counts were quantified by liquid scintillation to assess incorporation of ^35^S-labeled amino acids into nascent proteins.

### Analysis of ISVP* generation

ISVPs (2×10^11^) were incubated with 300 mM CsCl at 32°C for 20 min, transferred to ice for 20 min, and incubated with 100 µg/ml trypsin at 4°C for various intervals. Trypsin digestion was terminated by addition of SDS-PAGE loading buffer and removal of the samples to dry ice. Samples were resolved by electrophoresis in 4–20% polyacrylamide gels and immunoblotted using a μ1-specific MAb.

### Measurement of thermal stability

ISVPs in virion storage buffer were incubated at various temperatures for 15 min and transferred to ice for 1 h. Residual infectivity in triplicate samples was assessed by plaque assay [59].

### Quantification of apoptosis by acridine orange staining

HeLa cells (5×10^4^) in 24-well plates were adsorbed with reovirus at room temperature for 1 h. The percentage of apoptotic cells after 48 h incubation was determined using acridine orange staining as described [17]. For each experiment, >200 cells were counted, and the percentage of cells exhibiting condensed chromatin was determined by epi-illumination fluorescence microscopy using a fluorescein filter set (Zeiss).

### Quantification of apoptosis by propidium iodide staining

HeLa cells (1×10^6^) in 6-well plates were adsorbed with reovirus at room temperature for 1 h, followed by incubation for 48 h. Cells were harvested and fixed in chilled 70% ethanol by overnight incubation at 4°C. The percentage of cells showing hypodiploid nuclei was assessed by flow cytometry following incubation of fixed cells with DNA staining solution (1 mg/ml sodium citrate, 0.3% TX-100, 100 µg/ml propidium iodide, and 50 µg/ml RNase A) for 30 min [66].

### Luciferase assays

293T cells in 24-well plates were transfected with 0.72 µg/well of an NF-κB reporter plasmid, which expresses firefly luciferase under NF-κB control (pNF-κB-Luc), and 0.08 µg/well of control plasmid pRenilla-Luc, which expresses Renilla luciferase constitutively, using Lipofectamine 2000. After incubation for 24 h, transfected cells were adsorbed with reovirus in serum-free medium at room temperature for 1 h, followed by incubation at 37°C in serum-containing medium for 24 h. Luciferase activity in the cultures was quantified using the Dual-Luciferase Assay Kit (Promega) according to the manufacturer's instructions.

### Analysis of cellular distribution of μ1

CV-1 cells grown on cover slips (Fisher) were infected with reovirus and incubated at 37°C for 48 h. Cells were fixed in 2% paraformaldehyde in PBS at room temperature for 10 min, washed three times with PBS, and permeabilized using PBS containing 1% bovine serum albumin (BSA) and 0.1% Triton X-100 (PBSA-T) or 0.5% saponin for 5 min. Cells were incubated with primary or secondary antibodies in PBSA-T or PBS containing 1% BSA and 0.05% saponin at room temperature for 25 to 40 min. Coverslips were washed three times in PBS between primary and secondary antibody incubations. Cell nuclei were labeled by incubation of coverslips with 300 nM 4′,6′-diamidino-2-phenylindole (DAPI) (Invitrogen). Coverslips were mounted on glass slides with Prolong reagent (Invitrogen). Fluorescence and phase-contrast images were obtained using a Nikon TE2000 inverted microscope equipped with a 60×1.4 NA oil objective with 1.5× optical zoom. Images were collected digitally using a Coolsnap HQ charge-coupled-device camera (Roper) and Openlab software (Improvision) and prepared for publication using Photoshop and Illustrator software (Adobe Systems).

### Infection of mice

Two-day-old ND4 Swiss Webster mice (Harlan) were inoculated intracranially with purified rsT3D, K594D, or I595K in a volume of 5 µl into the left cerebral hemisphere using a Hamilton syringe and 30-gauge needle [9]. For analysis of viral virulence, mice were monitored for survival and symptoms of neurological disease for 21 d. Mice were euthanized when they were found to be moribund (defined by rapid or shallow breathing, lethargy, or paralysis). For analysis of virus growth, mice were euthanized at various intervals following inoculation, and brains were collected into 1 ml of PBS and homogenized by freezing, thawing, and sonication. Viral titers in brain homogenates were determined by plaque assay [59]. Animal husbandry and experimental procedures were performed in accordance with Public Health Service policy and approved by the Vanderbilt University School of Medicine Institutional Animal Care and Use Committee.

### Histology of mouse brains

Two-day-old ND4 Swiss Webster mice were inoculated intracranially with rsT3D, K594D, or I595K. Mice were euthanized, brains were resected, and brain tissues were fixed overnight in 10% formalin, followed by 70% ethanol. Fixed organs were embedded in paraffin, and 6-µm histological sections were prepared. Consecutively obtained sections were stained with H&E for evaluation of histopathologic changes or processed for immunohistochemical detection of reovirus protein or activated caspase-3 [11].

### Assessment of apoptosis in brain homogenates

Two-day-old ND4 Swiss Webster mice were inoculated intracranially with purified rsT3D, K594D, or I595K. Mice were euthanized at various intervals following inoculation, and brains were collected into 1 ml of PBS and homogenized by freezing, thawing, and sonication. Brain homogenates were diluted 10-fold in 1× RIPA, and 10–20 µg of protein extract was resolved by electrophoresis in 4–20% polyacrylamide gels and transferred to nitrocellulose membranes. Membranes were blocked for at least 1 h in Tris-buffered saline (TBS, 50 mM Tris [pH 7.5], 150 mM NaCl) containing 5% milk or 2.5% BSA and incubated with primary antibodies diluted 1∶500 (for the activated form of caspase-3) or 1∶2000 (for actin) in blocking buffer (TBS containing milk or BSA) at room temperature for 1 h. Membranes were washed three times for 10 min each with washing buffer (TBS containing 0.1% Tween-20) and incubated with Alexa Fluor 680- or Alexa Fluor 750-conjugated goat anti-rabbit (for caspase-3) or donkey anti-goat (for actin) Ig, respectively, diluted 1∶10 000 in blocking buffer. Membranes were washed three times and scanned using a LiCor Odyssey Infrared Imager. Intensities of pixels within the caspase-3 and actin bands were quantified using Odyssey software.
